# An Evaluation of the Significance of Work-Related Influence Factors on Fitness and the Development of Medical and Orthopaedic Conditions in Military Executives

**DOI:** 10.1155/2016/3929104

**Published:** 2016-09-27

**Authors:** Christoph Schulze, Michael Becker, Susanne Finze, Christoph Holtherm, Jens Hinder, Andreas Lison

**Affiliations:** ^1^Department of Orthopaedics, University Medicine Rostock, Doberaner Str. 142, 18057 Rostock, Germany; ^2^Bundeswehr Centre of Sports Medicine, Dr.-Rau-Allee 32, 48231 Warendorf, Germany

## Abstract

Occupational health promotion is an effective tool to improve the state of health of employees. As part of occupational health promotion in the German Bundeswehr, top-ranking military executives are offered a medical examination and training programme. Health-related data is collected as a basis for training and lifestyle counselling. This data was subjected to a retrospective evaluation in order to identify occupational risk factors and their correlation with cardiovascular resilience, trunk strength, and the development of orthopaedic and internal disorders. A total of 122 military executives (all male, age 54.6 ± 4.2 years) answered a questionnaire aimed at evaluating private and occupational stress factors. The medical history was followed by a medical and orthopaedic examination involving a lactate performance test (treadmill or bicycle ergometry) and an isometric trunk strength measurement. The data obtained was then statistically evaluated. For military executives, work-related travelling and commuting involve a high risk of medical and orthopaedic conditions. Regular exercise leads to improved fitness levels. In order to prevent medical problems, military executives working long hours should regularly take part in fitness and weight training under professional instructions.

## 1. Introduction

Occupational health promotion is getting more and more important. An increasing number of occupational health promotion projects in large and small businesses can be observed [1]. The fundaments of occupational health promotion are occupational medicine and participation of executives and management as well as health care [2]. Of great importance is the prevention of stress, addiction, nutrition, and physical activity [2]. Besides the occupational risk factors, further conditions have to be considered. Education level and social status influence the presence of certain risk factors for the development of diseases [3]. Executives in private companies and state organisations experience certain stress factors, both physically and mentally [4]. This results in particular health risks that differ from those faced by ordinary employees [5]. Blood pressure is a particularly significant parameter in this context. Positions involving a high level of personal responsibility have proven to contribute less to high blood pressure [6]. Incidence depends on age, sex, and BMI, with older employees in positions of high responsibility being more likely to develop cardiovascular diseases [6]. Physical fitness is an important parameter for coping with demands [7]. During working hours, executives in the army engage in less physical activity than other military personnel. During leisure time, on the other hand, the activity level of executive personnel is considerably higher [8]. This trend is also common in civilian companies [9]. Alongside physical resilience, mental resilience also plays an important role for military personnel. Mental resilience can be impaired by stress factors such as deployments abroad [10]. Long working hours also influence the development of cardiovascular diseases [11]. Lactate performance tests are one method of measuring physical resilience [12]. Strong trunk musculature decreases the likelihood of developing back pain [13]. A good method for determining trunk strength is to measure its isokinetic properties [14].

The availability of a workplace advisory service for employees with health problems proved to have positive effects on the employees' professional achievements and wellbeing [15]. This principle forms the basis for a range of seminars available for executives in the German Army. This is the reason why our centre established a seminar that combines presentations on health topics, medical examinations, and personal training. The purpose of the seminars is to lay the foundations for the participant's own health and for that of their subordinates. Following a thorough analysis of their own health situation, participants receive medical advice aimed at optimising physical and mental fitness. This retrospective study is designed to establish the degree to which the development of diseases and physical fitness are linked to occupational stress caused by deployments abroad, travel on official business, exercise, and commuting.

## 2. Material and Methods

In total, 122 male subjects took part in the prevention programme for executives during the study period from 2008 to 2012. All participants gave their written consent for scientific evaluation and publication of the results. The average age was 54.6 ± 4.2 years. The average BMI was 26.6 ± 2.8 kg/m^2^ and the average waist circumference was 95.9 ± 8.2 cm. Prior to equipment-based testing, the subjects were given a standardised questionnaire on the following points: occupation, weekly working hours, alcohol consumption, smoking habits, commuting activity (way to work, daily or weekly driving home, and time of deployment), exercise (hours per week during work and during leisure time and kind of exercises), mental stress, previous internal or orthopaedic disorders, and medications taken. This was followed by a nutritional analysis based on the* Freiburger Ernährungsprotokoll* (Freiburg Dietary Record). The subjects then provided a blood sample to determine the parameters HbA1c, blood count, ALAT, ASAT, *ɣ*-GT, sodium, potassium, creatinine, urea, blood glucose, total cholesterol, HDL cholesterol, and LDL cholesterol.

### 2.1. Lactate Performance Test

For the performance test, 86 patients used a stationary bicycle (Ergometrics 900, company: ErgoLine, Germany) and 36 patients used a treadmill (ELG 70/200, company: Woodway, Germany; ECG, company: Spacelabs Healthcare, Hawthorne, California, USA). The choice of exercise equipment was based on whether subjects preferred running or other types of sports. For bicycle ergometry, a stress profile was chosen which was started at an individual level, with the intensity increasing by 50 watts (W) every 3 minutes. For the treadmill ergometry, the stress profile was also started at an individual level and the speed increased by 1 km/h every 3 minutes. Measurement was discontinued when the patient became fatigued and refused to go on testing or showed signs of ischaemia in the ECG. Lactate measurement was performed before the exercise, at the end of each 3-minute period of exercise, and during the recovery phase. Blood samples were taken from the ear. The earlobe was pricked with a lancet (safety lancet, prick depth 1.8 mm, Sarstedt, Nümbrecht, Germany) and a drop of blood was collected by means of manual compression. The first drop of blood was discarded and the second was collected in a capillary tube (heparin-coated end-to-end capillary, EKF, Magdeburg, Germany). This blood sample was stored in an analysis kit (glucose and lactate haemolysis solution, EKF, Magdeburg, Germany). Lactate concentration was then established using a spectrometer (device: Biosen S-Line: EKF; Magdeburg, Germany). The data obtained was evaluated using Winlactat software (version V 4.6.3.21, Mesics GmbH, Münster, Germany). Performance or speed at 4 mmol/L lactate (*p*_4 mmolLactat_; *v*_4 mmolLactat_) and maximum performance or speed (*p*_max_; *v*_max_) were chosen as comparable parameters for evaluation. Following an individual performance analysis, the subjects received sports medical advice.

### 2.2. Isokinetic Measurement of Trunk Muscle Strength

Trunk muscle strength was measured using the IsoMed 2000 (D&R Ferstl GmbH; Hemau, Germany). Extension and flexion strength were established according to the manufacturers' recommendations and compared with standard values. The quotient of force (*F*) of extension and flexion was established so as to determine any muscular imbalance in the trunk musculature. On the basis of this parameter, a course of training was recommended for stabilising the trunk.

### 2.3. Statistical Evaluation

A descriptive data analysis was conducted for all groups (establishment of minimum, maximum, mean, and median). Group comparison was conducted by means of ANOVA with subsequent post hoc LSD analysis. The significance level was set at *p* < 0.05. The Mann-Whitney *U* test, also called the Wilcoxon rank-sum test, was used to conduct pairwise comparisons for dependent variables when comparing the subject's current weight with his weight upon joining the army. The significance level was determined at *p* < 0.05. For the correlation analysis, the correlation coefficient was determined in accordance with Pearson's *r*. *p* < 0.05 was specified as having a two-tailed significance level.

## 3. Results

### 3.1. State of Health of the Subject Group

Within the 122 subjects, we obtained 58 cases of hypercholesterolaemia, 38 cases of hypertension, 5 cases of diabetes mellitus, and 17 cases of hyperuricaemia. A total of 111 subjects reported orthopaedic health problems, with back pain being the most common complaint with 66 subjects. Overexertion of the musculoskeletal system (*N* = 39) and arthrosis of large joints (*N* = 6) were also specified. The development of orthopaedic conditions had a negative effect on physical fitness (*r* = −0.413; *p* = 0.012).

### 3.2. Comparison of the Fitness of the Various Rank Categories

Of the 122 officers examined, 55 were senior officers, 66 were generals, and 1 was a captain. To examine the differences within the rank categories, the generals were compared with the senior officers. Bicycle ergometry was chosen for 37 senior officers and 48 generals while 18 senior officers and 18 generals performed treadmill ergometry. No significant difference was established, but generals tended to be fitter than senior officers despite the fact that the former were significantly older (*p*_4 mmol-L_: senior officers: 157.73 W, generals: 170.2 W; *p* = 0.099; *p*_max_ senior officers: 217.30 W, generals: 226.42 W; *p* = 0.379).

### 3.3. Changes in Body Weight (Since Joining the German Bundeswehr)

Information available on current weight and weight upon joining the Bundeswehr was available for 116 subjects. Their average body weight on joining the Bundeswehr was 74.1 ± 7.1 kg. Their average body weight at the beginning of the preventive healthcare seminar was 87.4 ± 10.4 kg. During the period of an average of 35.6 ± 4.2 years of service, body weight had increased significantly by 13.3 ± 16.8 kg (*p* < 0.001). High weight upon joining the Bundeswehr correlated with high weight (*r* = 0.637; *p* < 0.001) and waist circumference (*r* = 0.637; *p* < 0.001) at the time of the study. An evaluation of the body mass index during that period could not be conducted because the height of the participants at the beginning of their service was not known.

### 3.4. Effect of Daily Working Hours on Physical Fitness

The average number of hours worked in a day was 10.57 hours. Subjects were divided into two groups. One group consisted of participants with an average number of working hours of ≤9.5 h/day (*N* = 79), which roughly corresponds to normal working time, and the other group consisted of participants who usually worked >9.5 h/day (*N* = 32). The remaining 11 patients provided no information about their working hours. No link was established between daily working hours and fitness (*p*_4 mmol-L_: 161.6 (±37.1) W to 166.23 (±37.5) W; *p* = 0.807). A high number of weekly working hours, however, correlated with an increased risk of developing a medical condition, in particular high blood pressure (*r* = 0.226; *p* = 0.017).

### 3.5. Effect of the Number of Deployments Abroad on Physical Fitness

Participants were divided into two groups: one in which the subjects had been on a Bundeswehr mission abroad and another in which they had not (*N* = 50 versus 62). Subjects with mission experience were significantly fitter than those without (bicycle ergometry: *p*_4 mmol-L_: 176.2 (±37.2) W versus 152.5 (±32.2) W; *p* = 0.002; *p*_max_: 242.33 (±48.1) W versus 204.4 (±41.9) W; *p* = 0.001). There was no evidence that participation in missions has an influence on the tendency to develop medical or musculoskeletal conditions.

### 3.6. Effect of Weekend Commuting on Physical Fitness

Participants were divided into two groups: one of subjects who commuted on weekends (*N* = 41) and one of subjects who did not (*N* = 71). The bicycle cardiac stress test revealed that, in terms of fitness, there was no significant difference between those subjects who commuted on weekends and those who did not (Table 1).

In the treadmill cardiac stress test, subjects who commuted on weekends on average revealed a lower level of fitness in comparison with subjects who did not commute on weekends (Table 1). The average maximum speeds tended to confirm this correlation (Table 1). Weekend commuting correlates with an increased number of daily working hours (*r* = 0.341; *p* < 0.001). Disorders of the musculoskeletal system tended to occur more frequently in weekend commuters (*r* = 0.181; *p* = 0.051). There was no evidence of an increased prevalence of diabetes mellitus or hypercholesterolaemia. In the group of weekend commuters, however, 21 participants had high blood pressure. In the comparison group, 16 subjects had high blood pressure. High blood pressure was significantly more common among weekend commuters (*p* = 0.002).

### 3.7. Effect of Increased Frequency of Work-Related Travel on Physical Fitness

Subjects were divided into two groups based on the indicated number of days they spent travelling for work: Group 1: moderate frequency of trips (≤35 days *n* = 53; 30 senior officers and 23 generals) and Group 2: high frequency of trips (>35 days *N* = 56; 22 senior officers and 33 generals). Evaluation of the treadmill cardiac stress test revealed a significantly lower level of performance among the group of subjects who travelled more frequently (Table 2). This trend was also apparent in the bicycle cardiac stress test but results were not significant (Table 2). In general, increased frequency of work-related travel has a negative effect on the fitness of executives and correlates with a high number of weekly working hours (*r* = 0.276; *p* = 0.004).

### 3.8. Effect of Exercise during Duty Hours on Physical Fitness

A total of 19 persons specified that they did not exercise during duty hours. However, 61 participants regularly exercised during working hours. No information was provided by 42 subjects. Those who exercised during working hours were significantly fitter than those who did not (Table 3). A high level of physical fitness correlated with strong trunk musculature, both in extension (*r* = 0.561; *p* = 0.037) and in flexion (*r* = 0.583; *p* = 0.029). Sporting military executives tended to have a stronger trunk musculature compared to nonsporting executives (Table 4).

## 4. Discussion

On the whole, medical problems were found to be less common among the examined sample than the normal population. A link was established between poor physical fitness and the rate of orthopaedic problems. While in Germany approximately 70% of men between the ages of 50 and 60 have high blood pressure, only 31% did so in our sample [16, 17]. The percentage of subjects with hypercholesterolaemia in our sample (48%) was higher than the national average of 33% [18, 19]. The incidence increases with age and decreasing level of education [20]. Our sample almost exclusively included participants aged 50 or older, but all of them had completed university education. This is combined with a higher social status and this suggests that the high proportion of participants with hypercholesterolaemia must have other causes [3]. The 4% proportion of diabetics in the sample, which is below the average of 15% in this age group, can be explained by the fact that early development of diabetes mellitus renders soldiers unfit for service and thus ineligible for promotion [20]. Approximately 60% of subjects experienced orthopaedic conditions, in particular lower back pain, which corresponds to the national average in the age group examined [20].

Analysis of performance capability revealed that top military executives were physically fitter than the military executive level below them, despite the fact that the top executives were considerably older. This suggests that the ability to advance to this echelon from the level below is partly subject to a certain level of physical fitness and that the ability to successfully cope with other stress factors is not enough on its own. It is likely that a good physical condition makes it easier to deal more effectively with the challenges of professional life. Most importantly, our study revealed that weekend commuting and work-related travel during duty hours have a negative effect on physical fitness. As civilian comparative studies have also shown, working hours as such have a less significant influence [21]. Military executives who had been on deployments abroad were fitter than those who had not. However, since deployments abroad involve certain basic physical fitness requirements, there were probably certain health restrictions present that prevented the latter from being deployed.

A significant starting point for taking action as part of professional preventive health care is the fact that the integration of sports into military duty helps improve physical fitness, thus addressing medical and orthopaedic conditions. This applies despite the fact that executives are most physically active outside of working hours because one of the aims of professional health promotion is to bring about a change in lifestyle [22]. On the whole, however, it is remarkable that military executives can gain an average of 14 kg of body weight over the years even though they are obligated by rules to maintain physical fitness and a good health status. Therefore, it is essential that the identified risk groups (commuters, personnel who travel frequently, and individuals with previous illnesses) can be reached with professional health promotion programs. Such programs have to be offered to motivate personnel to be more active and health oriented. We proved, after all, that duty sports increase fitness levels. In addition, well-trained trunk musculature has a preventive effect on musculoskeletal conditions [13]. When starting an exercise programme, soldiers should initially receive instructions in order to increase effectiveness and prevent an increase in the number of individuals suffering from excessive strain as a result of incorrect training [15]. These instructions can also be given as part of a seminar, such as the one offered in our centre. Military executives are the preferred target group in occupational health promotion because they can act as a positive example and it is their responsibility to plan physical activity during duty [23]. So it is important to offer health education to this target group to spread the information of occupational health promotion to subordinated personnel.

It must be mentioned, however, that there are limitations in our study. First of all, there is the lack of a significant number of revaluations that confirm our statements. The number of 122 participants is unexceptional. There may be a bias in the selection of our participants, because they took part in our program with the motivation to improve their state of health. So it may be possible that military executives with less motivation to learn how to improve the personal state of health could have worse results.

## 5. Conclusion

Among the Bundeswehr executive personnel in our sample, medical conditions are somewhat rarer than among the population average of the age group under examination. However, work-related travel and commuting in particular could be risk factors for physical inactivity, which, however, should be countered effectively and positively with weight and endurance training as part of professional health promotion.

## Figures and Tables

**Table 1:** Comparison of the fitness data of commuters and noncommuters. All data are presented as mean ± standard deviation.

Participants (bicycle/treadmill)	*p*_4 mmol-L_ in W	*p*_max_ in W	*v*_4 mmol-L_ in km/h	*v*_max_ in km/h
Commuters (*N* = 29/12)	167.9 (±31.2)	228.5 (±48.0)	9.7 (±1.4)	12.2 (±1.4)
Noncommuters (*N* = 51/20)	164.4 (±40.1)	223.8 (±50.5)	10.8 (±1.5)	13.2 (±1.6)
*p*	0.509	0.584	0.035	0.058

**Table 2:** Fitness data of military executives who travel frequently and moderately often: a comparison. All data are presented as mean ± standard deviation.

Participants (bicycle/treadmill)	*p*_4 mmol-L_ in W	*p*_max_ in W	*v*_4 mmol-L_ in km/h	*v*_max_ in km/h
Group 1 (*N* = 39/14)	169.9 (±38.5)	232.7 (±52.6)	11.1 (±1.4)	13.4 (±1.5)
Group 2 (*N* = 39/17)	163.2 (±35.7)	221.9 (±44.4)	9.9 (±1.5)	12.6 (±1.7)
*p*	0.418	0.383	0.029	0.115

**Table 3:** Comparison of fitness data between sporting and nonsporting military executives. All data are presented as mean ± standard deviation.

Subjects (bicycle/treadmill)	*p*_4 mmol-L_ in W	*p*_max_ in W	*v*_4 mmol-L_ in km/h	*v*_max_ in km/h
Exercise (*N* = 40/21)	169.1 (±37.8)	233.1 (±50.4)	10.9 (±1.6)	13.5 (±1.6)
No exercise (*N* = 17/2)	138.8 (±23.1)	178.1 (±33.1)	8.0 (±1.0)	11.1 (±0.4)
*p*	0.003	<0.001	0.029	0.037

**Table 4:** Comparison of strength of the trunk muscles measured during isokinetic testing between sporting and non sporting military executives (only 50 participants took part in this investigation). All data are presented as mean ± standard deviation.

Subjects	Flexion (*F* in Nm)	Extension (*F* in Nm)
No Sports (*N* = 3)	1.67	2.33
Sports (*N* = 47)	1.74	3.04
*p* value	0.848	0.062
